# Rhinitis, sinusitis and ocular disease – 2092. The MSNOT-20 questionnaire: repeatability and disease analysis of rhinitis/rhinosinusitis

**DOI:** 10.1186/1939-4551-6-S1-P170

**Published:** 2013-04-23

**Authors:** Amtul Salam Sami, Masood Malik, Mohammed Amjad, Peter Howarth

**Affiliations:** 1 Ent and Allergy Department, London, UK; 2 Services Hospital, Ent Department, Pakistan; 3 Southampton, UK

## Background

Sinonasal disease is prevalent and impacts on quality of life, the Modified SNOT-20 (MSNOT-20) questionnaire aims to provide a complete assessment of rhinitis/rhinosinusitis and its impact. Following a pilot study evaluation, the main project assessed the prevalence of rhinitis/rhinosinusitis in a community based survey in Farnborough, UK. A sub group was invited for clinical examination and repeatability of MSNOT-20.

Further repeatability was carried out through a study of self-referral patients to the ENT department of Services Hospital, Lahore, Pakistan. This study aimed to obtain the current burden of rhinitis/rhinosinusitis in these patients and subsequent disease course.

## Methods

Following a successful pilot study comparing disease with non-disease, 2000 postal questionnaires were sent to randomly selected adults. A sub group of the sample was invited to the hospital for clinical examination and for the repeatability of MSNOT-20.

The Lahore study looked at 200 patients who completed the MSNOT-20 questionnaire on initial presentation and, over the course of 3 consultations, were assessed for disease response to provided therapy by completing MSNOT-20 at each point. For unresponsive cases to conventional therapy, blood tests were used to identify the aetiology of disease.

## Results

The pilot project showed significant differences (p<0.001) between disease and non-disease. Analysis of responses allowed a discriminating score of ≥2 (on a 0-5 scale) being an abnormal response. The Farnborough study identified 32% of respondents (79.8% response rate) as disease with significant correlations between disease domain and the 4 other domains (paranasal [sinus and ear], sleep, social and emotional).

Analysis of the Lahore study found 25% of the self-referral population suffered from rhinitis /rhinosinusitis, 5% of patients had a form of surgical intervention ranging from antral wash out to endoscopic sinus surgery, 2% had associated pathologies in the form of deflected nasal septum, nasal polyp or antrochoanal polyp.

## Conclusions

MSNOT-20 questionnaire has identified a high prevalence of nasal and paranasal problems within the community and in self referrals with significant associated co-morbidity. The MSNOT-20 has proven to be a valid, reliable and repeatable disease specific quality of life questionnaire able to evaluate the presence, severity and assessment of response to treatment.

